# Coping strategies of families of persons with learning disability in Imo state of Nigeria

**DOI:** 10.1186/s41043-019-0168-2

**Published:** 2019-03-27

**Authors:** Ngozi E. Chukwu, Uzoma O. Okoye, Nkechi G. Onyeneho, Joseph C. Okeibunor

**Affiliations:** 1 Department of Social Work, University of Nigeria, Nsukka, Enugu State Nigeria; 2 Department of Sociology/Anthropology, University of Nigeria, Nsukka, Enugu State Nigeria; 3 Takemi Program in International Health, Harvard T.H. Chan School of Public Health, Harvard University, Boston, MA USA; 4 WHO Regional Office for Africa, Brazzaville, Congo

**Keywords:** Learning disability, Coping, Coping strategies, Families, Nigeria

## Abstract

**Background:**

Coping with a relative with a learning disability could be a stressful experience for family members. The present study is aimed at exploring the coping strategies adopted by families in trying to make meaning of their situation.

**Method:**

A qualitative study design using focus group discussions (FGDs) was adopted. Ten FGD sessions were held with family members of persons with a learning disability.

**Results:**

Findings revealed patterns of family coping to include problem-focused, emotion-focused, and spiritual/religious-focused. Also, coping responses to a learning disability varied based on the level of information available to families about the condition of their relative. In some cases, interspousal relationship was strained due to stress.

**Conclusion:**

It was recommended that families of persons with a learning disability need social support and professional help from social workers to facilitate the adoption of more positive-oriented coping strategies by family members.

## Introduction

A learning disability is a state of arrested or incomplete development of mind which includes significant or severe impairment of intelligence and social functioning. It affects between 1 and 2.5% of the general population in the Western world [1], but in sub-Saharan Africa, where malnutrition is prevalent, the prevalence of learning disability puts at 2.9% or more of the population or moderate [2, 3]. A more recent systematic review puts the prevalence to be as high as 19.8% (95% CI, 18.8–20.7%) [4]. The risk factors include micronutrient deficiencies, iodine and iron deficiency, and anaemia [2, 5].

Learning disability usually leads to major functional impairment and lifelong need for support and interventions which most often can only be given by family members. The diagnosis of a learning disability has a profound impact not only on the person diagnosed, but also on the entire family. It is an event which changes and impacts all facets of family life. According to Jovanova and Radojichikj [6], a child with a learning disability requires a lot more time and attention from its parent in comparison to other children. Therefore, a learning disability is of significant concern for families. Literature has indicated that raising a child who has been diagnosed with a learning disability is a daunting and exhausting task [7–11]). Families can easily become overwhelmed by the process of finding and funding appropriate services and keeping many appointments. Some families may feel isolated, lonely, and confused [12]. At a time when families need more support than ever, support may be difficult to find. Relationships may be awkward and strained as spouses blame each other or other relatives for being responsible for the condition of the family member with a learning disability.

Sometimes, families are faced with the stress of continually witnessing their loved ones struggle to complete everyday tasks, social interactions, and education. The stress is also heightened by social stigmatisation as well as isolation from neighbours and community members due to ignorance and poor definition of learning disability in the cultural setting [13]. There is also the stress of caring for a family member with a learning disability which most often could be a 24-h job. All these have emotional, physical, and financial implications for family members. The ability to respond positively to these stresses despite exposure to risk or adversity is a key feature in most definitions of resilience [14, 15]. Resilience in this context can thus be defined as a psychological process that facilitates healthy functioning in response to intense life stressors. Since caring experiences are likely to continue for an extended period of time, resilience is likely to be necessary on an ongoing basis, rather than in response to a single traumatic event [16].

The resiliency model of family stress and adjustment which is based on a family systems approach has been used as a way of explaining why some families remain resilient in the face of adversity [17]. This model divides a family’s response to life changes into two phases, specifically the adjustment phase and the adaptation phase. Adjustment is a short-term response to the stressor involving minor changes in the family. However, if the demands of the stressor exceed the family’s coping strategies, more substantial changes are required. The adaptation phase describes the family process as involving changes in established roles, rules, goals, and/or patterns of interaction. Therefore, this model is of the view that the families’ adjustment to the stressors associated with a learning disability depends on the families’ characteristic mode of behaviour and their problem-solving and coping responses. The condition of a learning disability is stressful not only for the individual but also for the family. This is especially the case during the period of transition of the person from childhood to adult life. This period could be occasions for further crisis, which can shake the stability of the family as a system especially if their problem-solving and coping responses are not adequate.

Coping according to Campbell [18] means an ability to adjust, adapt, and meet a challenge successfully. It also entails contending or dealing successfully with a challenging event. Folkman and Lazarus [19] noted that coping means when one constantly changes his/her cognitive and behavioural efforts in order to manage some specific external or internal demands that have been judged as tasking or exceeding the resources of the person. On the other hand, Tennen et al. [20] are of the view that the central function of coping is the reduction of tension and the restoration of equilibrium. Hagemann [21] distinguishes between two types of coping. These are coping that is directed at managing or altering the problem causing the distress (problem-focused) and coping that is directed at regulating emotional response to the problem (emotion-focused).

Coping, however, does not occur in a vacuum. Rather, individuals use various strategies to enable them cope with stressful situations. Coping strategies refer to the specific efforts, both behavioural and cognitive, that people adopt to master, tolerate, reduce, or minimise stressful events [22]. Two major categories of coping strategies are widely recognised: problem-focused coping strategies (efforts to do something active to alleviate stressful circumstances) and emotion-focused coping strategies (efforts to regulate the emotional consequences of stressful or potentially stressful events) [22, 23]. However, a third category referred to as spiritual/religious-focused coping strategies (finding meaning and purpose to adversity through a strong relationship with God) has been added [24].

Abundant research literature on coping strategies is available in the context of a wide range of illnesses and disabilities [25–29]. In the context of a general learning disability, some relatively recent attention has been paid to the issue of coping [30–35]. To date, however, research findings have not focused on coping strategies adopted by families of persons with learning disabilities in Nigeria using qualitative data. This paper aims at identifying the coping strategies that family members use in trying to face the challenges of coping with having a family member with a learning disability through quotes from discussions. Identifying such coping strategies may facilitate the development of more effective social work services to families with persons with learning disabilities (PLDs) in Nigeria.

## Methods

### Study design

The study was exploratory. It adopted a cross-sectional approach using qualitative methods of inquiry, based on focus group discussion designs, to allow a description of family coping strategies to learning disabilities in a family member in Imo state.

### Study area

The study was conducted in Imo state in southeast Nigeria, which is the homeland of the Igbo. Imo state comprises of three senatorial zones, namely Owerri, Okigwe, and Orlu, that constitute the bases of political representation in the central legislature. Together, these three senatorial zones are delineated into a total of 27 local government areas (LGAs), where each LGA is an administrative entity created by Nigeria’s political authorities. The Igbo are deeply religious and generally hold the belief that causally, a congenital condition like learning disability derives from the spiritual realm is still quite strong. The methodological assumption, in a setting with such socio-cultural belief may impact on how learning disability is perceived by the study particpants and by extension the coping of family members.

### Participants

Participants were 107 (parents and siblings) of persons with a learning disability (PLDs) (18 fathers, 23 mothers, 30 brothers, and 36 sisters) who sat through focused group discussions (FGDs). The inclusion criteria in the sample included the presence of at least one person with a learning disability in the household and the attainment of age 10 by the siblings in households with PLD.

### Materials and procedure

The study adopted a qualitative methodology for data gathering and analysis because the concomitant free-flowing format that ensues enables relevant insights into how people make sense of their experiences about issues [36]. That rationale underscored why the FGD was adopted as the instrument for data collection. The FGD was effectively utilised to elicit information on a range of issues including lived experiences with persons with a learning disability and the different forms of coping strategies that family members deploy in their efforts to effectively grapple with the challenges of living with a family member with a learning disability.

For the needs of the study, one LGA was purposively selected from each of the three senatorial zones of the state for the FGDs. Using simple random sampling by balloting, the researchers selected four autonomous communities from each of the three LGAs. The outcome is a total of 12 study communities where we located the households with persons with a learning disability, and these were the households that furnished the 107 participants in the study. Since learning disability does not obtain in a systematic manner in society, one would discern that it required rigour and diligence on the part of study authors to identify and locate families that met the study criteria for the selection of participants in each location.

Thus, the snowball sampling technique also known as “chain referencing sampling” or “respondent-driven sampling” was employed in the study, because the study authors believe that persons with disability constitute a typical example of a “hidden population” [37]. The first household in each community was located with the help of ward leaders. Subsequently, each case helped to identify other cases and location of the households. From these households, we purposively selected two or three respondents (two siblings and a parent) aged 10 years and above. The siblings were matched in terms of a male and a female. However, where we could not get the matching two, one sibling was selected.

Ten FGDs were conducted as follows: 2 with fathers, 2 with mothers, 3 with sisters, and 3 with brothers. Each session was made up of between 9 and 12 participants. The FGDs were conducted in settings that were both conducive and preventive of interference by non-participants. Each discussion lasted an average of one and a half hours. All discussions were conducted in Igbo language and taped with an audio recording device. The lead author conducted the interviews with the assistance of undergraduates of the University of Nigeria, Nsukka, who functioned as note takers. One of the main qualifying criteria for each undergraduate who was co-opted to assist in the study is their fluency in the version of the dialect of the Igbo language spoken in Imo state. The Ethical Committee of the University of Nigeria Teaching Hospital, Ituku-Ozalla, Enugu, which is also an integral part of the University of Nigeria, Nsukka, where the study authors are employed, approved the study protocol. Informed verbal consent was also obtained from all the participants for the audio recording of discussions.

The outcomes of the FGDs were transcribed into the English language by two graduate students from the linguistic department of the University of Nigeria, Nsukka. Both graduate students are fluent in the dialect of the Igbo language spoken in Imo state. To ensure additional quality control, the transcripts were carefully read by one of our colleagues in the social work programme who is also fluent in the dialect of the Igbo language spoken in Imo state. On yet another level of quality control, the transcripts were read and studied multiple times by the study authors using strategies of both content and narrative analysis. Through these processes, the study authors were able to highlight prominent themes in the transcripts and also pay the necessary attention to both the overt and latent issues evident in the contents of the narratives.

## Results

Their ages ranged from 13 to 67 years, with a mean age of 45.5 years. About 50% of the parents were civil servants while the rest were farmers, traders, and artisans. No parent was unemployed. All the siblings had completed at least 6 years of schooling while about 27% of the siblings had completed 16 years of schooling.

Three major themes were identified through the analysis of the qualitative data in terms of coping strategies of family members. These were the problem-focused, emotion-focused, and spiritual/religious-focused coping strategies.

### Problem-focused coping strategies

Some of the respondents indicated that they adopted the problem-focused coping strategy by seeking information in order to understand the condition of the family member with a learning disability. They expressed that getting information from professionals and television programmes helped them gain knowledge on the different aspects of learning disability. A father of a person with a learning disability stated, “I try to get information from professionals like doctors and nurses. I feel these people are in a better position to tell us how to go along”. Another father opined:

When we noticed that my daughter was slow and not growing mentally as her age mates were, I took her to FMC [Federal Medical Center], Owerri. We saw a paediatrician who referred us to a brain expert. The brain expert after conducting some tests confirmed my worst fears, that she has a learning disability… I then started searching the internet to know more about the condition and how to proceed. This search to be honest has helped me. Hmm, in fact not only me but my entire family! We no longer feel embarrassed. We now take her to church and even for family outings. In fact, we pet her a lot and encourage her… praising her when she improves in an activity and also patient for her shortcomings.

Other participants opined that getting information on learning disability helped them to adopt more problem-focused coping strategies. According to them, information from television programmes helped them gain knowledge on the different aspects of learning disability, what to expect and how to teach them basic skills like hygiene, feeding, safety, and communication. This knowledge helped them realise that there is nothing wrong with them as a family. They also stated that it was through the information from such programmes that they came to know what causes learning disability, thereby debunking the superstitious myth that it is a curse. They expressed that knowledge from such programmes also helped them overcome unnecessary shame when in public with their family member with a learning disability. A sister had this to say “I have learnt different ways to cope with my sister’s condition from the Digital Satellite Television [DSTV] program we watched. I now take her along to visit my friends without feeling ashamed. I am now so close to her now”. A mother has this to say in the following narrative:

In our own case, there was a time a DSTV program did a documentary on families with such persons. Every member of my family watched that program. This program was very educative. It helped us realise that we are not alone… that some families have the same problem…even white people. It was from it that we now know what to expect and how to teach him activities of daily living.

Adequate knowledge and information from professionals helped families in making the decision to place the family member with a learning disability in institutional care. Contrary to the view (due to lack of information) that there will never be an improvement on the condition of a person with a learning disability due to ignorance, some families with adequate knowledge who sought information got to know of the availability of institutions for care and training of persons with a learning disability. A mother who had a daughter with a learning disability indicated that putting her in an institution in Enugu for care and training has helped immensely in her daughter’s learning of activities of daily living. This has also helped the family as a whole to cope with her condition. In her own words:

Before we took her to the Therapeutic Center in Enugu, she was very hyper active and could not do anything for herself, but this last time she came back, they have trained her. She now stays at a place and is learning some activities of daily living. The burden of caring for her has lessened to a large degree.

It is important to note that these participants were from relative highly educated elite families. They live in houses with modern communication facilities like the DSTV. In terms of religion, they were however mixed, but predominantly Christians. These were rather scientific unlike their less educated and poorer counterparts who were largely emotional.

### Emotion-focused coping strategies

Some of the participants become very emotional as the discussions go on, from their facial expression as well as body language when the question of what causes learning disability was raised. There were indications of letting off steam on other people through suspicions and blaming of each other especially the spouses. Blames ranged from the spouses accusing each other of either infidelity or curse due to the spouse’s previous wrongdoing. This is best illustrated in the response of a mother and father of a person with a learning disability. The mother said:

I learnt that my husband gave his mother so much trouble and heart break as a young boy and his mother cursed him, telling him that she will come as a useless daughter to him. Look at me now. I am suffering because of something I have no hand in. (Shakes her head). Had I known, I would not have married into this family (Breaks down and sobs)

While the father had this to say:

It is the fault of my wife. Her waywardness is what brought this problem to my family. Do I talk to her? She is the one suffering with his care. My only regret is the shame and ridicule it is putting my family through.

Feelings of resentment, bitterness, and fear of the unknown have negative effects on interspousal relationship. This was expressed when the issue of having more children was discussed. A mother expressed that she is afraid of getting pregnant again because the curse following her husband might lead her to give birth to another child with a learning disability. According to her:

I don’t even sleep in the same room with him. I am afraid of getting pregnant and getting another child like this one. I have suffered a lot and to make matters worse he does not help me in caring for this one. Is not me and him? Maybe his curse is still following him.

Some participants feel that the family member with a learning disability gets preferential treatment over other family members. This view was mostly expressed by other siblings who feel that their mother, in particular, is so attached to their sibling with a learning disability. They feel that he/she gets more attention from their mother more than the other children. A brother of a person with a learning disability opined that “I don’t know why our mother is so attached to him. She has no time for those of who are healthy. She makes sure he gets and takes any good thing before all of us”.

Some participants also suggested other strategies that they use. Their views ranged from pretending the person with a learning disability does not exist to adopting attitudes like overeating. Others include keeping away from people or locking and keeping the person with a learning disability out of public view. According to a female sibling:

I am embarrassed by this condition in my family but what can I do? The worst part is that he is the first person to appear once there is a visitor in this house. I am already 32 years old and any suitor who comes runs away immediately he notices the condition of my brother. I am indeed depressed especially since my last suitor left me when he found out that I had a sibling with a learning disability. I have started eating large amount of food and I am keeping out of public view. Ask people who knew me I was not like this initially

Another male sibling also said:

We lock him up to prevent people from seeing him. Also he does not go anywhere with us. People are always laughing at us because of the way he behaves. It is very shameful. He just has to be at home locked up.

A mother with a son with a learning disability sums her coping strategy of resignation to fate this way:

I have resigned to fate. I try to pretend that this does not worry me but when I am alone it disturbs me. I physically make myself find a quiet place. I remove myself from most of the gatherings of women in this community. You know the way women are. I don’t want anybody to make a side comment that will upset me…I am resigned to my fate. They feel that it is my fault and they blame me because I did not take my antenatal serious when I was pregnant with him.

Other participants especially females indicated that use of social support from friends and professionals helped. They opined that finding someone to listen to them and giving them words of encouragement helps. This is demonstrated by the following narrative from a female participant:

I am not ashamed to talk to people about my child’s condition. At times when I speak to them, some of them are very sympathetic, offering me words of encouragement. Some even give me addresses of places to visit with my son. It makes me not to feel isolated. Look at the way you people came to this our community and have been talking and teaching us things we did not know about this problem. If people like you people come from time to time to talk to us it will help give us hope that people feel our pains.

### Spiritual/religious-focused coping strategies

This coping strategy was adopted by family members who held strong religious beliefs. Families who were active in the church received ministrations from the church, which enabled them to cope with stress and have a positive outlook for their child. Coping responses included seeking help from their religious and spiritual community. They got counselling as well as referral to institutions for the care of persons with a learning disability. According to one female participant:

I have been getting comfort and understanding from our parish priest. He helped to link us to the center at Oguta. We went there to see if we can get some help. We were advised to place him in that institution for training but we could not afford the cost. It is very expensive. I am just a labourer. I weed people’s farms and cannot afford the money and all the things they listed for us to bring

Living with a family member with a learning disability is a situation that tests the spiritual beliefs of people especially in a cultural setting where it is believed that the condition is a curse from the gods. Some family members, out of feelings of being punished or abandoned by God and resignation to the will of God, resort to searching for spiritual cleansing. The following illustrative quote from a mother whose child has a learning disability indicated that some of them use various coping mechanisms such as resigning to the will of God as Christians:

As a Christian, I strongly believe that if God has not willed this, my family will not be in this situation. One of the things keeping me comforted is my religious belief. Where haven’t we gone? At first, we were told to visit a herbalist because it was seen as a curse or witchcraft. When this was not working, we resorted to seeing powerful pastors. We have taken him to crusade for deliverance. We have seen many pastors but no change. I have prayed, fasted and cried and have decided to turn the situation over to God.

Another mother said:

Maybe I am being punished by God for my sins. If not, how come that among all my brothers and sisters that got married, I am the only person that has a child with a learning disability? Let God just do with me what he wants

Family members also indicated another spiritual path which they used as a coping strategy. The path some families chose was going to see a native (traditional) doctor. This choice of coping strategy according to them was helpful since it is their belief that learning disability is caused by witchcraft. They were of the view that the visit to the native doctor was helpful in calming the hyperactivity and other disturbing behaviours of the person with a developmental disability. As a father opined:

This my son here, used to be very restless. He used to be very destructive. In fact, there was a time he pushed his younger sister and she fell into hot oil. We were told that he is possessed by an evil spirit…this problem was the result of the quarrel my wife had with my uncle’s wife when she was pregnant. Because we knew that this problem has some witchcraft attached to it, we had to see the native doctor for help. He gave us some liquid to add to the water for his bathing and some oil to rub on his body. Ever since then, he is much calmer and behaves more like a sensible human being.

## Discussion and conclusion

Coping with any form of disability in the family has been a source of concern for psychologists, social workers, and researchers. This has informed investigations in the social sciences into how family members of persons with a learning disability react/cope with the condition. This is because the response of family members to the problem is of crucial importance to their coping as well as the future wellbeing of the person with a learning disability. The findings of this study revealed the various coping strategies the families of persons with a learning disability adopt. Three major coping strategies emerged. These were problem-focused, emotion-focused, and spiritual/religious-focused coping strategies. Some sub-themes/variants of these coping strategies also emerged. These include denial/passive coping, empowerment coping, social withdrawal, and acceptance coping.

Some of these findings support previous research that families who demonstrate optimistic or hopeful outlooks and family belief systems are more resilient. These families more cognitively process information and cope better rather than reacting in highly emotional ways [38–40]. The finding that usage of positive coping strategies that were more problem-focused for dealing with the stress of having a family member with a learning disability is helpful in reducing the stress is similar to what was found by Jones and Passey [41]. Adoption of more problem-focused coping strategies was helpful to family members to reformulate the disability of the family member in a more positive way, thereby becoming more competent and having better family adaptation [42]. Use of appropriate information and advice on learning disabilities from professionals and media as indicated from the findings are necessary positive coping strategies that are problem-focused. This coping strategy was very helpful in empowering families with adequate knowledge to help both themselves and their family member with a learning disability. It has been suggested in previous research that information is a very important determinant for coping with stressful situation in families [43]. Findings from this study also revealed that religious/spiritual coping strategies were used in a positive way by some families. Family members who were active in church activities used religious coping in a more positive way and had a more positive outlook towards the condition of their relative with a learning disability. It further helped them make meaning and develop more acceptance of the situation.

Furthermore, results indicated that some families are in denial and adopted avoidant coping strategies. They try to cope by pretending the problem does not exist, blaming each other, or being withdrawn socially. The use of avoidant coping strategy by families according to literature implies some families try to cope with the problem by creating a surreal image whether positive or negative or they ignore the disabilities of their family member [43]. Also, some husbands and wives blamed each other which in turn affected their marital union.

Coping strategies varied based on educational level, level of information, social status, sex, and social support available to families. It was observed that families who narrated that the information they got from professionals and through watching DSTV programmes helped them in understanding the condition were more educated and financially stable. This is because not many families subscribe to satellite television which is usually not cheap. Such families also are more likely to have the economic power to place the family member with a learning disability in institutional care. Females, especially mothers, found emotion and spiritual/religious coping strategies very helpful. Females equally were more likely to use social support both formal and informal forms.

## Implications for practice, teaching, and future research

These findings are important when one considers their practical significance and implications for future research. Families of persons with a learning disability experience a lot of emotional and psychological stress which makes coping difficult. Most of their reactions are borne out of ignorance and lack of information. This leads some of them to adopt coping strategies that are not positive-oriented. Looking at the practice side, findings from our study support the need for practitioners to educate parents on the best coping strategy that will help them cope with children with a learning disability. By doing so, social workers will have the opportunity of making an impact in the lives of their clients and also help those families that trade blames and are in denial.

Our findings also revealed that some of the participants obtained useful information from the news media that helped them to cope. This has implication for practice in that the social work bodies and associations can organise enlightenment campaigns in using various media to pass information to the populace about learning disability especially letting people know that such children are not a curse for an evil deed. This study therefore concluded that social work professionals will be helpful in giving families information that will help their understanding of learning disability so that they can adopt more positive-oriented coping strategies.

Social support programmes in the form of social services are essential for families of children with a learning disability. These support programmes should be more family-oriented, that is, focusing on the needs of all the family members instead of focusing on the needs of the person with a learning disability. Such services, which will be managed by social workers, should utilise and encourage religious practices of the family as a resource. Other appropriate strength-based family support services should also be considered. It is therefore recommended that social workers and other helping professionals should be employed in the communities for this service. They will give family members proper information on learning disability which will help reduce their reliance on avoidant coping and increase the use of positive coping strategies.

In the realm of research, it may be important for new researches to examine in-depth the relationship between such factors as superstitions, low level of education/knowledge and religion, on the one hand, and coping ability of parents of children with a learning disability. This is because in the present study, some of the participants stated that religion helped them not only to cope but also to give meaning and acceptance to the problem, but our findings may have been more conclusive with a larger sample. This was particularly true for the two.

Christian mothers relied on miraculous healing from God. Future research efforts that will focus exclusively on gender and coping strategies with a larger sample also are needed as they will help us to understand the relationship between gender and coping strategies adopted by men and women. Interestingly, the women in our study used more emotion and spiritual/religious coping strategies than men.

This study was conducted in Imo state, Nigeria, which is predominantly Igbo speaking and where most of the people are adherents of the Christian religion, while only very insignificant few persons are adherents of African Traditional Religion (ATR). One of the interpretive implications of those facts is that the results from this study cannot be used to make generalisations for Nigeria, which British colonial intervention was carved out of the distinct peoples that inhabit this portion of the greater Niger basin. Noteworthy in this regard as well is that some of Nigeria’s distinct peoples are Muslims. Future research of this nature needs to be conducted in Nigeria’s other administrative locations that encompass different sociocultural peculiarities to ascertain whatever differences that the outcome would hold.

## Abbreviations

ATR: African Traditional Religion
DSTV: Digital satellite television
FGD: Focus group discussion
LD: Learning disability
LGA: Local government area
PLD: Person with a learning disability
